# ARMC5 Primary Bilateral Macronodular Adrenal Hyperplasia Associated with a Meningioma: A Family Report

**DOI:** 10.1155/2020/8848151

**Published:** 2020-09-02

**Authors:** M. J. Ferreira, J. Pedro, D. Salazar, C. Costa, J. Aragão Rodrigues, M. M. Costa, A. Grangeia, J. L. Castedo, D. Carvalho

**Affiliations:** ^1^Department of Endocrinology, Diabetes and Metabolism of Centro Hospitalar Universitário de São João, Porto, Portugal; ^2^Faculty of Medicine of the Universidade do Porto, Porto, Portugal; ^3^Instituto de Investigação e Inovação em Saúde, Universidade do Porto, Porto, Portugal; ^4^Portuguese Institute of Oncology, Porto, Portugal; ^5^Department of Pathology of Centro Hospitalar Universitário de São João, Porto, Portugal; ^6^Department of Endocrinology, Diabetes and Metabolism of Hospital Garcia de Orta, Porto, Portugal; ^7^Department of Genetics of Centro Hospitalar Universitário de São João, Porto, Portugal

## Abstract

Primary bilateral adrenal macronodular hyperplasia is characterized by functioning adrenal macronodules and variable cortisol secretion. Familial clustering suggests a genetic cause that has been confirmed with the identification of some genetic mutations, including inactivating germline mutations, in *armadillo repeat containing 5 (ARMC5)* gene. The identification of the pathogenic variant enables the physician to identify and treat these patients earlier and more effectively. It has also been noticed that patients with germline causative variants show a different clinical spectrum, presenting specific clinical characteristics, as the association with the presence of meningiomas.

## 1. Introduction

Primary bilateral macronodular adrenal hyperplasia (PBMAH) is a rare cause of Cushing's syndrome, presenting multiple benign nodules in both adrenal cortexes [1]. In contrast to micronodular adrenal hyperplasia, adrenal nodules observed in patients with PBMAH are typically larger than 1 cm and detectable by abdominal imaging [2]. PBMAH may lead to overt Cushing's syndrome and long-term consequences of cortisol excess are frequent despite appropriate treatment. This might be due to diagnostic delay, as mild clinical presentation is often observed [2–4]. PBMAH is most frequently diagnosed in patients between 40 and 70 years of age and frequently in the course of investigation of an adrenal incidentaloma [2]. Because of its clinical variability, the prevalence of this disease is not well-established, but is thought to represent less than 2% of all endogenous cases of Cushing's syndrome [1, 5].

The pathophysiological process that leads to PBMAH has not been fully clarified [6–8]. Steroidogenesis has been shown to be relatively inefficient in PBMAH, which explains the indolent natural history of the disease, and the late manifestation of clinical hypercortisolism, present only after severe and irregular enlargement of the adrenal glands [2].

Although previously considered an ACTH-independent Cushing syndrome, it now appears that cortisol secretion by the adrenals is regulated by corticotropin, which is locally produced by a subpopulation of steroidogenic cells in the hyperplastic adrenals [7, 9]. The abnormal expression of ACTH by these cells seems to represent a secondary pathophysiological process that is common to diverse molecular defects and is seen in both sporadic and familial cases [7]. In most patients with PBMAH, there are also aberrant receptors that regulate steroidogenesis by mimicking the cellular events that are triggered normally by the ACTH receptor [10]. As so, the secretion of cortisol often involves stimulation of ectopic membrane receptors that shows hyperactivity or paradoxical stimulation to glucose-dependent insulinotropic peptide, beta-adrenergic receptors, serotonin, glucagon, and probably angiotensin; as well as eutopic receptors such as those for vasopressin, LH/human chorionic gonadotropin, or serotonin [11–13].

Germline and somatic mutations were also reported to be involved in the mechanism causing PBMAH. Genes associated with PBMAH include ARMC5 (*armadillo repeatcontaining 5)*, PDE11A (*Phosphodiesterase 11A)*, APC (*Adenomatous polyposis coli*), and FH (*Fumarate hydratase*) [6]. ARMC5 mutations were found to be the most frequently mutated gene in PBMAH. Different studies show prevalences of ARMC5 mutations ranging from 19.6% to 55%, mostly sporadic cases [3, 4, 14]. ARMC5 likely functions as a tumor suppressor gene, and somatic mutations have been detected in addition to the germline mutations in patients with PBMAH, supporting the “two-hit” model for ARMC5 as a tumor suppressor gene [3, 15, 16]. The occurrence of intracranial meningiomas has also been associated with PBMAH, suggesting a possible role of ARMC5 for the development of other neoplasia [17].

## 2. Case Presentation

**Figure 1:**
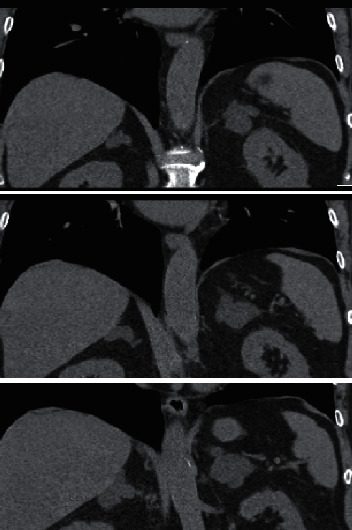
Adrenal CT scan of the index patient.

A 64-year-old man, referred to endocrinology due to bilateral adrenal incidentalomas on a CT scan, measuring 4 × 4.3 cm on the left and 4.3 × 4.2 cm on the right side, with characteristics of adenoma/hyperplasia (Figure 1). He had assumed type 2 diabetes mellitus, hypertension, and dyslipidemia for about 4 years and had suffered an acute myocardial infarction 3 years before. He was being treated with four different antihypertensive medications (bisoprolol, spironolactone, olmesartan, and amlodipine), as well as gliclazide and simvastatin. Physical examination revealed thin and dry skin, obesity with centripetal fat distribution, multiple ecchymosis, and facial erythrosis.

He had a 41-year-old son diagnosed with schizophrenia and an apparently healthy 37-year-old daughter. No known familial relevant diseases.

His laboratory workup revealed hypercortisolism: failure to suppress on the 1 mg overnight dexamethasone suppression test-cortisol 27.6 *µ*g/dL-and on the low-dose dexamethasone suppression test (0.5 mg every 6 h for 48 h) end of the test-cortisol 24 *µ*g/dL-associated to morning ACTH < 1 ng/L.

He underwent stimulation tests with Tetracosactide, LHRH (luteinizing hormone-releasing hormone), TRH (thyrotropin-releasing hormone), vasopressin, metoclopramide, glucagon, and upright posture test, which were overall negative, with the exception of partial response to the upright posture test (cortisol variation of 42.5%).

**Figure 2:**
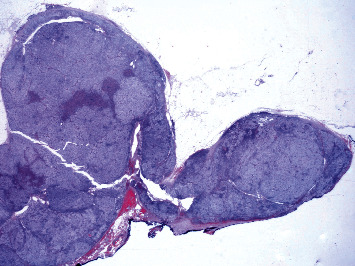
(H&E, original magnification x2.5)-Histologically, there is evidence of adrenal cortex thickening, with architectural disorganization and multinodular appearance.

**Figure 3:**
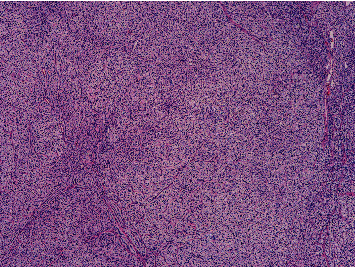
(H&E, original magnification x4 and x10)-There is ill-defined multinodular hyperplasia of the cortical fasciculata layer of the adrenal tissue.

**Figure 4:**
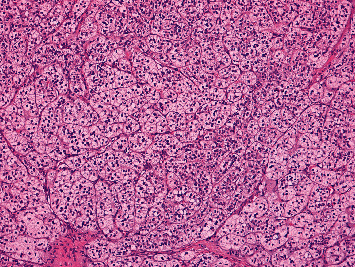
H&E, original magnification x4 and x10)-There is ill-defined multinodular hyperplasia of the cortical fasciculata layer of the adrenal tissue.

**Figure 5:**
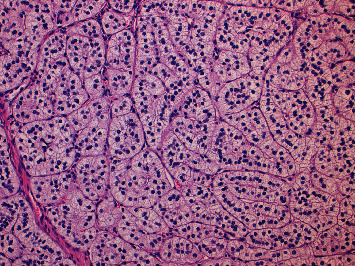
(H&E, original magnification x20)-The hyperplastic cortical fasciculata layer is characterized by a large number of cord organized cells with well-defined membranes, clear and vacuolated cytoplasm and, small nuclei.

It was then decided to proceed bilateral adrenalectomy. However, due to surgical complications, only right adrenalectomy was performed. On gross examination, a right adrenalectomy specimen was surrounded by fat with 94,6 g with 8,5 × 4,3 × 2 cm adrenal gland in size was removed. The adrenal gland was compact and yellow, with nodular appearance on section. On histological examination, the adrenal tissue had architectural disorganization associated with ill-defined multinodular hyperplasia of the cortical fasciculata layer. These aspects were consistent with adrenal nodular hyperplasia (Figures 2–5).

The patient now remains free of hypercortisolism, with normal blood pressure and glucose control, with no need of medication.

**Figure 6:**
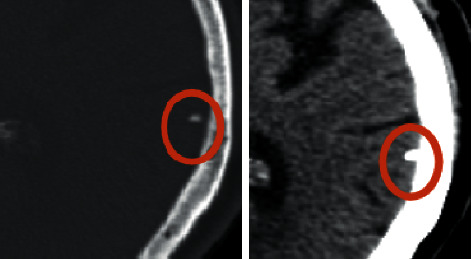
Cerebral CT scan of the index patient.

A molecular study in DNA extracted from peripheral blood leucocyte and in the adrenal gland revealed the presence in heterozygosity of the pathogenic mutation c.1379T < C in the ARMC5 gene, which encodes the protein variant p(Leu460Pro). Due to this finding, the patient underwent a cerebral CT scan, which showed a meningioma in the posterior left temporal convexity (Figure 6).

**Figure 7:**
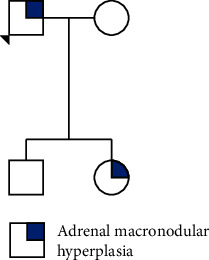
Family tree: 1. Proband, 2. affected daughter, 3. healthy son. Circles: females; squares: males.

Genetic testing was also performed on the daughter and son, which revealed the same pathogenic mutation. They were also tested for hypercortisolism and underwent adrenal and cerebral CT scan (Figure 7).

His son had no physical signs of hypercortisolism. His blood tests did not reveal any finding suggesting hypercortisolism: 1 mg overnight dexamethasone suppression test–cortisol 0.8 *µ*g/dL, morning ACTH 18.6 ng/L. His adrenal and cerebral CT scans showed no abnormalities. His daughter had a history of easy bruising, but no other signs suggestive of hypercortisolism. As with her brother, her blood tests did not reveal hypercortisolism: 1 mg overnight dexamethasone suppression test–cortisol 1.0 *µ*g/dL and morning ACTH 19.1 ng/L; and her cerebral CT scan was normal. Her adrenal CT scan, however, showed enlargement of the left adrenal gland, with aspects suggestive of nodular hyperplasia, with a more evident and nodular area of 14 mm in diameter.

Both of them are currently under annual active surveillance, with yearly evaluation for hypercortisolism.

## 3. Discussion

PBMAH is a heterogeneous and complex disorder with varying degrees of hypercortisolism (from subclinical to overt Cushing's syndrome), variable size and number of nodules, and expression of illegitimate membrane receptors. The frequency of such illegitimate receptors in PBMAH is high, varying from 77% to 87% [2, 18]. This illegitimate receptors are G protein-coupled receptors stimulating adenylyl cyclase, inducing cortisol secretion [19]. PBMAH is also associated with paracrine intraadrenal ACTH secretion. This ectopic ACTH is secreted by adrenocortical cells, located in the subcapsular region and in hyperplasic nodules which express both proconvertase 1, the ACTH-precursor cleavage enzyme, and the usual enzymatic resources of cells capable of steroidogenesis. This condition is slightly more prevalent in women, but the reason is not clear [19].

ARMC5 gene mutations has been identified in a large number of patients and familial cases with PBMAH [3, 6–8, 17]. ARMC5 is a cytosolic protein with no enzymatic activity, containing 7 armadillo domains and one BTB domain towards its C-terminus, which allows its dimerization or trimerization [20]. More proteins are known to contain armadillo domains, including the well-known beta-catenin. Armadillo-containing proteins are involved in many different functions, including T-cell, neural tube, and adrenal cortex development as well as tumor suppression. ARMC5 inactivation leads to decreased steroidogenesis. Despite the reduced cortisol secretion capacity of each cell, progressive hypercortisolism results from the increased number of adrenocortical cells, explaining the slow and progressive development of Cushing syndrome [3]. The ARMC5 antiapototic effect is responsible for the growth of bilateral masses [2, 8, 16]. The presence or absence of ARMC5 mutations may be associated with diverse phenotypes [16]. Patients with ARMC5 mutation tend to present a more severe presentation, with younger age at diagnosis, larger adrenal nodules and a higher prevalence of clinical CS [2, 16]. These patients also seem to show a higher abnormal cortisol response during the upright posture test, which was also seen in our index patient [2]. In addition, ARMC5-mutated patients have hypertension more often than wild-type patients [16, 21]. In fact, ARMC5 mutations are also responsible for some cases of primary hyperaldosteronism, mainly in Afro-American patients [21]. Intracranial meningiomas have also been described in patients harboring (p.Ala110fs*∗*9) ARMC5 mutation with PBMAH, while others lacking these mutations do not develop this condition [7, 16, 17, 21]. Our patient also had a meningioma, despite having a different mutation than described (c.1379T < C, p.Leu460Pro). It seems that some degree of genotype-phenotype correlation is present [16].

Bilateral adrenalectomy has been considered the treatment of choice for PBMAH, but it leads to permanent adrenal insufficiency, requiring lifelong glucocorticoid and mineralocorticoid replacement and impaired quality of life [22]. Unilateral adrenalectomy has recently been proposed as an alternative in selected patients, with some advocating for the removal of the larger gland, especially in older patients [23]. Recently the use of mifepristone has also been proposed [24].

No specific follow-up protocol has been proposed to mutation carriers without Cushing syndrome diagnosis. In our center, we perform a yearly biochemical evaluation.

## 4. Conclusion

All patients with PBMAH or suspected of having the disease should be tested, and once a germline mutation is identified, first degree patients should also undergo genetic testing and counseling [7]. Considering the insidious nature of this disease and the known deleterious effects of hypercortisolism, this will allow physicians to timely identify and treat this patients.

Our findings correlate with the literature available, and they support the idea of a new “syndrome” which may include the association between PBMAH and meningioma. More studies are needed to confirm this hypothesis.
